# Genome sequence of *Paracoccus sandarakinolimnes* ME4, a pigmented, aerobic bacterium isolated from freshwater

**DOI:** 10.17912/micropub.biology.001660

**Published:** 2025-07-18

**Authors:** Victoria Abramczuk, Virginia Saionz, Maria Estevez, Emily Stowe

**Affiliations:** 1 Department of Biology, Bucknell University, Lewisburg, Pennsylvania, United States

## Abstract

*Paracoccus* is a metabolically diverse genus found in a range of habitats. Here we describe *Paracoccus sandarakinolimnes* ME4, a carotenoid-producing, aerobic bacterium isolated from freshwater. The genome contains 4,061,481 bases in five contigs with 4,028 protein-coding genes, 106 tRNA and rRNA genes, and 35 pseudogenes.

**Figure 1. Colony morphology, nutrient requirements and genome structure of Paracoccus sandarakinolimnes:**
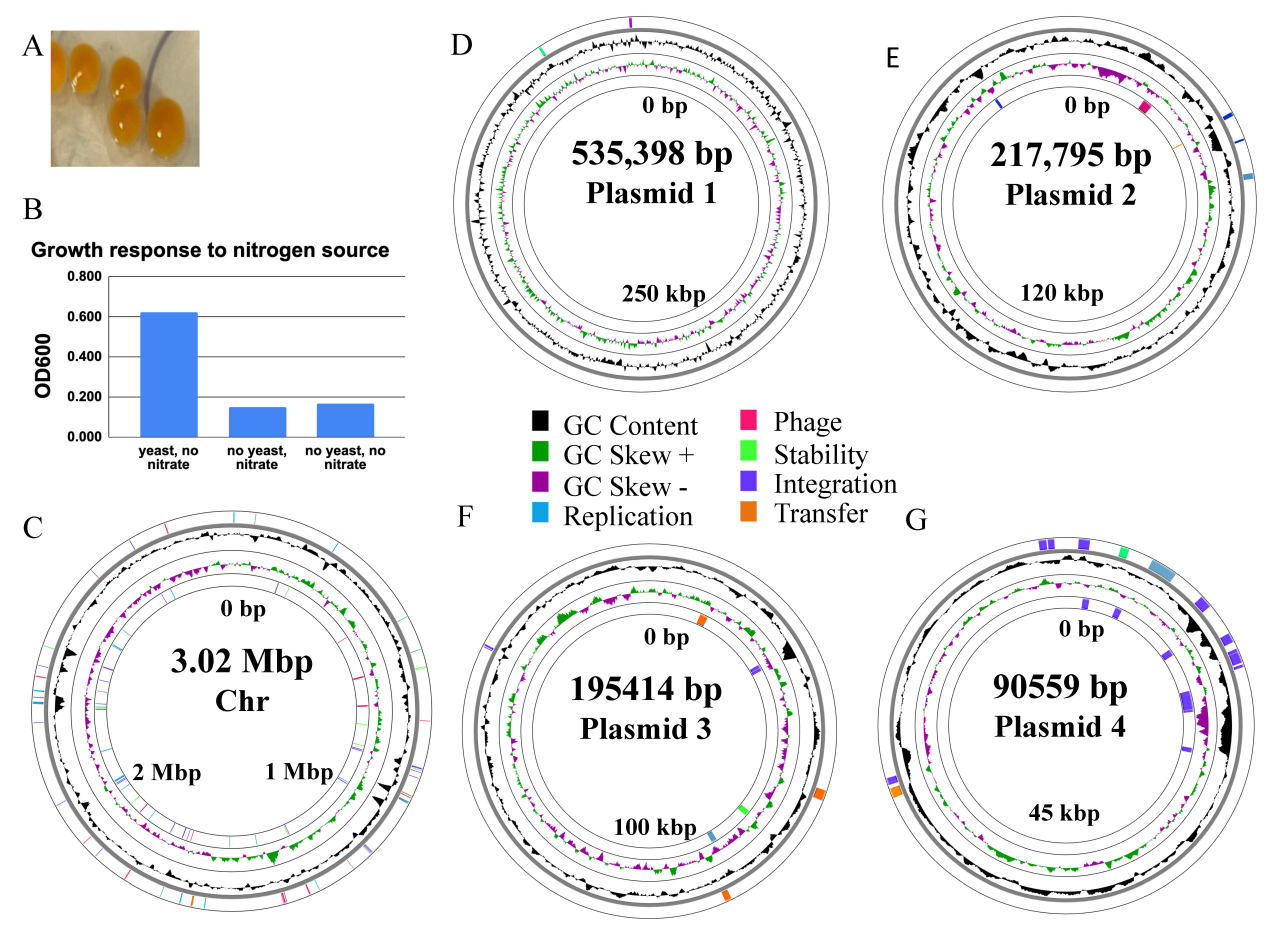
(A) Colonies of ME4 grown on BG11 media supplemented with mannitol and yeast extract (B) OD600 of liquid cultures grown for 24 hours at 25°C in BG11 media with mannitol media with just yeast extract added, just sodium nitrate added, neither yeast extract nor nitrate added. (C) Chromosome (Chr) and (D-G) putative plasmid maps of *P.* *sandarakinolimnes* ME4 visualized by Proksee (Grant et al., 2023) using the Proksee GC content tool 1.0.2, and mobile genetic elements identified by mobileOG-db (beatrix-1.6)(Brown et al., 2022). Total length of each DNA element is within the circle; 0bp and approximate additional sites are also noted. The outermost and innermost rings of the maps represent mobile genetic elements whose products might be required for maintenance and transfer of plasmids (Brown et al., 2022) in the positive and negative reading frames respectively. Dark blue regions include sequences involved with integration and excision of plasmids into and out of the chromosome including transposases. The pink region in E encodes a homolog of TssH, part of the TypeIV secretion system. The orange region in E contains a relaxase domain while in F and G these regions encode ABC transporter subunits. Light green regions as in G encode genes related to plasmid stability and defense such as VapBC toxin/antitoxin system (Arcus et al., 2011). Light blue regions includes genes like *repAB* used for plasmid replication during cell division (Peterson et al., 2009). The second ring from the outside indicates the GC content (black) and the third ring the positive (green) or negative (purple) skewing from the average GC content.

## Description

Recent work by Louca et al. (2019) estimates the global diversity of prokaryotes at 0.8 to 1.6 million different OTU (operational taxonomic units). A vanishingly small number of these organisms have been characterized individually. We isolated a novel bacterium (hereafter called ME4) from a freshwater pond on BG11 media (Rippka et al., 1979) supplemented with mannitol while attempting to isolate nitrogen fixing organisms. Though ME4 lacks nitrogen fixation ability, we maintained the organism due to its orange pigmentation, likely a carotenoid ( Figure 1A ). Carotenoids are studied for their potential health benefits and as additives in food, animal feed, and cosmetics (Demmig-Adams et al., 2020, Sereti et al., 2025). Although carotenoids can be synthetically manufactured, interest remains in their biosynthesis by microorganisms (Conradie et al., 2018, Ram et al., 2020, Naik and Gupte, 2024). Many *Paracoccus* species have previously been explored as sources of carotenoids (Conradie et al., 2018, Maj et al., 2020, Naik and Gupte, 2024, Hwang et. al, 2024).

Intrigued by the pigmentation of ME4 and interested in determining if its carotenoid production differed from other known *Paracoccus* carotenoid producing strains, we carried out additional phenotypic and genetic analyses. ME4 is obligately aerobic and mesophilic, but grows poorly above 30 °C. ME4 requires an organic nitrogen source as indicated by reduced growth when supplied solely with sodium nitrate ( Figure 1B ). Preliminary analysis of growth on BIOLOG GENIII plates indicates ME4 can use a wide range of carbon sources, tolerates mildly acidic conditions (pH6 not pH 5) and increased salinity (1% and 4% NaCl not 8% NaCl). BLAST analysis of the PCR amplified 16S gene product indicates a 99.09% similarity to *Paracoccus marcusii* MH1(NR044922.1) (Altschul et al, 1990; Weisburg et al., 1991; Harker et al., 1998). *Paracoccus* is a taxonomically diverse and widespread genus with few characterized freshwater species (Puri et al., 2021; Puri et al., 2022, Hollensteiner et al., 2023).

We sequenced the genome of ME4 using combined Illumina and Oxford Nanopore technologies. The assembled genome is 4,061,481 bases in five contigs ( Figure 1C- G) with a GC content of 68.51%. NCBI Prokaryotic Genome Annotation v6.7 identified 4,169 total genes, 4,028 protein-coding genes, 106 tRNA and rRNA genes, and 35 pseudogenes (Tatusova et al., 2016). Although not shared across the genus, multiple species of *Paracoccus* such as *P. carotinifaciens, P. haeundaensis,* and *P. marcusii* can synthesize carotenoids and have been considered for optimization of bacterial carotenoid production for the food, cosmetic and pharmaceutical industries (Conradie et al., 2018, Maj et al., 2020, Ram et al., 2020, Naik and Gupte, 2024, Maharjan and Kim, 2025). ME4 contains a *crtWZYIBE* operon (accession numbers WP_411837231-WP_411837236) which, given the orange pigmentation of the colonies, indicates the ability to generate a range of carotenoids from geranyl diphosphate (Choi et al., 2007). Contigs 2-5 contain homologs of plasmid replication genes *repAB* supporting their identification as plasmids (Petersen et al., 2009). Despite 99.09% identity to the 16S rRNA gene for *P. marcusii* MH1, the highest relationship observed via KBase FastANI v0.1.3 (Arkin et al., 2018, Jain et al., 2018) analysis to 44 *Paracoccus* complete genomes (Genbank October 2024, Clark et al, 2016) is 88.86% ( *P. marcusii* MBLB0836 (GCA_028621715)). This value is below the accepted ANI species delimitation threshold (Richter and Rosselló-Móra 2009, Hollensteiner et al., 2023) thus we chose the name *Paracoccus sandarakinolimnes* for ME4 to reflect both its color and habitat. Sandarakinos is Greek for orange-colored and limnes in Greek refers to a marshy or watery place. Continuing work with ME4 includes identification of carotenoids produced, optimization of carotenoid production and confirmation of plasmid identity.

## Methods

We isolated *Paracoccus sandarakinolimnes* ME4 from a small, spring-fed pond on the Bucknell University Natural Area (41.020402, -76.747581) in 2021. ME4 is an orange pigmented bacterium initially isolated on modified 1.5% agar solidified nitrate-free BG11 media supplemented with 1% mannitol at 22℃ (Rippka et al., 1979). Axenic cultures were obtained by streaking on the same media supplemented with 0.5g/L yeast extract. Growth tests were performed in liquid media from single isolated colonies at 25°C. Cultures were grown for 48 hours, diluted back to OD600 0.1 in nitrate free BG11 + 1% mannitol with either yeast extract (0.5 g/L), sodium nitrate (17.6 mM) or neither as a source of nitrogen. OD600 was subsequently measured at 24 hours. ME4 was streaked onto BUG media (biolog.com) and grown at 25°C for three days. A single isolated colony was resuspended into inoculating fluid A (biolog.com), aliquoted into a GENIII Aerobic Bacteria 2.08.09 plate (biolog.com) and incubated at 22°C for three days. Growth was indicated by color production and was assessed by eye. Anaerobic conditions were created in a Thermo Scientific™ AnaeroPack™ 2.5L Rectangular Jar (R685025) using Thermo Scientific™ AnaeroPack™-Anaero Anaerobic Gas Generator (R681001). A single colony was streaked onto a BG11 plus mannitol and yeast plate and incubated at 22°C in the anaerobic jar; no growth was observed after one week.

We used ZYMO Quick DNA Bacterial Fungal MidiPrep kit (D6501) to isolate DNA from cells grown at 25℃ in liquid BG11 plus mannitol and yeast media. Combined Illumina and Oxford Nanopore sequencing was completed at SeqCenter (seqcenter.com) using the Illumina DNA Prep kit (tagmentation-based) and Genomic DNA by Ligation kit (SQK-LSK109). Quality control and adapter trimming was performed with bcl2fastq (2.20.0.445) (https://support.illumina.com/sequencing/sequencing_software/bcl2fastq-conversion-software.html) and porechop (0.2.3_seqan2.1.1) (https://github.com/rrwick/Porechop) for Illumina and ONT sequencing respectively. Illumina sequencing yielded 3946349 read pairs and 1285094409 bases. We obtained 239704 nanopore trimmed reads with 1247546496 nanopore trimmed bases. Hybrid assembly with Illumina and ONT reads was performed with Unicycler v. 2021 (0.4.8) (Wick et al, 2017). Assembly statistics were recorded with QUAST (5.0.2) (Gurevich et al., 2013). Initial assembly annotation was performed with Prokka (1.14.5) using --rfam parameters (Seemann, 2011). Default parameters were used except where noted. The total assembled length is 4,061,481 bases in five contigs. Genome coverage is 623X. The N50 is 3,022,315 (putative chromosome). Contigs 2-5 are putative plasmids: contig 2 (535,398 bp), contig 3 (217,795 bp), contig 4 (195,414 bp), and contig 5 (90,399 bp).

The name sandarkinolimnes was developed in consultation with the Bucknell Ancient Greek 102 course in Spring 2025 under the direction of Dr. Stephanie Larson. Sandarakinos is Greek for orange-colored and though not common, was used by Herodotus. It has been used more recently in the bacterial name *Sandaracinomonas limnophila* (Chen et al., 2020) and the name of the Orange Skunk clown fish, *Amphiprion sandaracinos* . Limnes in Greek refers to a marshy or watery place.

## Reagents

Data availability statement

This Whole Genome Shotgun project has been deposited in GenBank with contig accession nos. CP151276.1, CP151277.1, CP151278.1, CP151279.1, and CP151280.1, Bioproject accession no. PRJNA1096720 and Biosample accession no. SAMN40760478 . The version described in this paper is the first version GCF_046736325.1. The organism is available from the corresponding author.
